# Squamous cell carcinoma of the oral cavity and the oropharynx in patients less than 40 years of age: a 20-year analysis

**DOI:** 10.1186/1758-3284-4-28

**Published:** 2012-05-30

**Authors:** Samuel E Udeabor, Majeed Rana, Gerd Wegener, Nils-Claudius Gellrich, André M Eckardt

**Affiliations:** 1 Department of Oral and Maxillofacial Surgery, University of Port Harcourt Teaching Hospital, Port Harcourt, Nigeria; 2 Department of Cranio-Maxillofacial Surgery, Hannover Medical School, Carl-Neuberg-Strasse 1, 30625, Hannover, Germany; 3 Clinical Tumor Registry, Hannover Medical School, Hannover, Alemanya

**Keywords:** Squamous cell carcinoma, Prognosis, Oral cavity, Oropharynx, Young adults

## Abstract

**Background:**

Squamous cell carcinoma mainly afflicts patients older than 40 years of age however, few cases are seen in younger patients. The aim of this study therefore was to determine the incidence of squamous cell carcinoma of the oral cavity and oropharynx in patients less than 40 years of age with a view to assessing the prognosis over a period of time.

**Methods:**

This was a 20 years retrospective review of patients who were histologically diagnosed with squamous cell carcinoma of the oral cavity and the oropharynx at the Department of Cranio-Maxillo-Facial Surgery of the Hannover Medical School, Germany and had not received treatment anywhere else. Records of these patients were analysed for age and sex distribution, tumour staging and differentiation, location, treatment given, recurrences and metastasis, time between diagnosis and death or last contact with patient, and possible cause of death. Comparisons were also made with patients older than 40 years of age.

**Results and discussion:**

A total of 977 patients treated for squamous cell carcinoma of the oral cavity and the oropharynx in the 20-year period of this study were included. Thirty eight (3.9 %) of the overall patient population were under 40 years of age. Among these, 30 (78.9%) were males and 8 (21.1%) were females. The incidence was highest in the 30–39 year age group accounting for 31 (81.6%) of the 38 patients. The moderately differentiated carcinoma was commonest (24; 63.2%). The floor of the mouth had the highest number of tumours (15; 39.5%), but none was seen in the oropharynx. Surgery alone was the main stay of treatment given to 26 (68.4%) patients. At the end of the study period, 13 (34.2%) patients had died of the tumour and the 5-year survival rate was 66.2%. In the older patient group (>40 years), 42.7% died from the tumour and the 5-year survival rate was 57.6%.

**Conclusion:**

The results from the present study showed that young adults may have a better prognosis especially in terms of long term overall survival from oral and oropharyngeal carcinoma.

## Background

Squamous cell carcinoma (SCC) in the head and neck region occurs primarily in the oral cavity and oropharynx and is generally regarded as a disease of the elderly [1]. Majority are seen in patients over the age of 40 years, whereas the tumour remains very uncommon among young adults [2]. A review of literature revealed that the incidence of SCC of the oral cavity (OC) and the oropharynx (OP) in young adults ranges between 1-6% [3-5] with the tongue frequently reported as the commonest location for this tumour[4].

It is logical to assume that the fewer cases of this tumour in young patients are due to the considerable shorter exposure period to the risk factors of alcohol and tobacco when compared to the elderly patients [3]. However, some cases have been reported in non-smoking, non-alcohol drinking youths which then raises the question as to other possible aetiological factors in this young population [6,7].

There is no agreement among various reports on the prognosis of SCC of the OC and OP in young adults. While some claim that there is no difference in prognosis between the disease in the elderly and the young adult patients [8,9], others report a worse prognosis in the young adults and therefore recommend a more aggressive management regime for this group [10].

We therefore present our experience in the management of patients with SCC of the OC and the OP over a 20-year period with particular attention to patients less than 40 years of age and also making comparisons to older patients above 40 years.

## Methods

The records of patients who were histologically diagnosed with squamous cell carcinoma of the oral cavity and the oropharynx at the Department of Cranio-Maxillo-Facial Surgery of the Hannover Medical School, Germany between 1980 and 1999 were retrospectively reviewed. Patients included in this study must not have been treated for their cancer anywhere else.

The data extracted were analysed for demographic information, tumour differentiation, size, location, treatment given, recurrences, second primaries and metastasis, time between diagnosis and death or last contact with patient, and possible cause of death. Patients were divided into 9 groups according to their ages at diagnosis: Group 1 (10–19 years); group 2 (20–29 years); group 3 (30–39 years); group 4 (40–49 years); group 5 (50–59 years); group 6 (60–69 years); group 7 (70–79 years); group 8 (80–89 years); and group 9 (90 years and above). TNM system was used for the tumour staging whereas the location was categorised using the International Classification of Diseases of the WHO into:

ICD-141 (Tongue),

ICD-143-143.13 (Mandibular and Maxillary mucosa),

ICD-144-144.9 (Floor of the mouth),

ICD-145-145.9 (Other parts of the mouth),

ICD-146-146.2 (Tonsillar region) and

ICD-146.6-146.9 (Oropharynx).

Treatment given to the patients was divided into surgery, radiotherapy, chemotherapy, hormone therapy, a combination of these or none. Statistical analysis was done using SPSS for windows (version 9.0) and the calculation of survival rates for the groups less than 40 years and those above 40 was done using the Kaplan-Meier estimates. Log-rank test was the statistical test used and a result is considered significant when p is < 0.05.

## Results

### Demographics

A total of 977 previously untreated patients, with histologically diagnosed SCC of the OC and the OP were seen within the period under review in this study. Male patients were 733 (75%), and female patients 244(25%) and the mean age for the general patients population was 57.6.

Thirty eight (3.9%) of the overall patient population were under 40 years of age, and 30 (78.9%) of them were males and 8 (21.1%) female. The youngest male patient at diagnosis was 24 years old and the youngest female patient 19 years. (Table 1)

**Table 1:** Age and gender distribution of the general patients population (n = 977)

**Age in years at diagnosis of SCC**	**Number of Patients**	**male female**		**percentage (%)**
15-19	1	0	1	0,1
20-29	6	4	2	0,6
30-39	31	26	5	3,2
40-49	222	182	40	22,7
50-59	358	289	69	36,6
60-69	211	154	57	21,6
70-79	104	57	47	10,6
80-89	43	20	23	4,4
≥ 90	1	1	0	0,1
**Total**	**977**	**733**	**244**	**100**
Average age in years	57,6	56,3	61,4	

### Tumour differentiation grading

Among the patients less than 40 years of age, 24 (63.2%) had moderately differentiated SCC, 7(18.4%) had well differentiated tumour and 5 (13.2%) had poorly differentiated tumour. Information regarding histological differentiation could not be found for 2 patients in this age group. Tables 2 and 3 show the histological differentiation in the general population and in those less than 40 years respectively.

**Table 2:** Histological grade of differentiation (all patients)

**Histological Differentiation**		
	**n**	**%**
1 well differentiated	141	14,4%
2 moderately differentiated	672	68,8%
3 poorly differentiated	105	10,7%
4 undifferentiated, anaplastic	2	0,2%
Value	920	94,2%
missing	57	5,8%
Total	977	100%

**Table 3:** Histological grade of differentiation (patients younger than 40 years)

**Histological Differentiation**		
	**n**	**%**
1 well differentiated	7	18,4%
2 moderately differentiated	24	63,2%
3 poorly differentiated	5	13,2%
Value	36	94,7%
Missing	2	5,3%
Total	38	100%

### Tumour location

The floor of the mouth was the commonest site of tumour occurrence both in the general population and in patients less than 40 years of age. This accounted for 42.2% in the general patient population and 39.5% in the group less than 40 years. Only in 1.3% of the overall patient population was the tumour located in the orophaynx and none was found in the oropharynx of the young patient group. (Tables 4 and 5)

**Table 4:** Tumour location for all patients

**Tumour Location** **(simplified according to ICD)**	**Number of Patients** **(n)**	**percentage** **(%)**
Tongue	220	22,5
Floor of the mouth	412	42,2
Other parts of the mouth	152	15,6
Mandibular and Maxillary mucosa	131	13,4
Tonsillar region	49	5,0
Oropharynx	13	1,3
**Total**	**977**	**100**

**Table 5:** Tumour location in patients younger than 40 years of age

**Tumour Location** **(simplified according to ICD)**	**Number of Patients (n)**	**males females**		**Percentage (%)**
Tongue	13	9	4	34,2
Floor of the mouth	15	13	2	39,5
Other parts of the mouth	3	2	1	7,9
Mandibular and Maxillary mucosa	4	3	1	10,5
Tonsillar region	3	3	0	7,9
Oropharynx	0	0	0	0
**Total**	**38**	**30**	**8**	**100**

### Tumour staging

**Figure 1:**
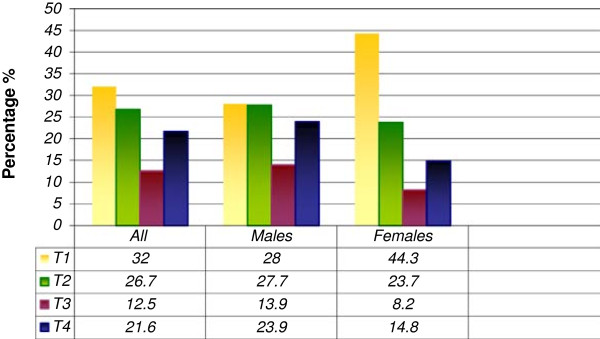
**T-stages (based on T1-T4) and sex of the patients (n = 977).** A comparison of men versus women using the log-rank test showed a highly significant result (p = 0.005).

**Figure 2:**
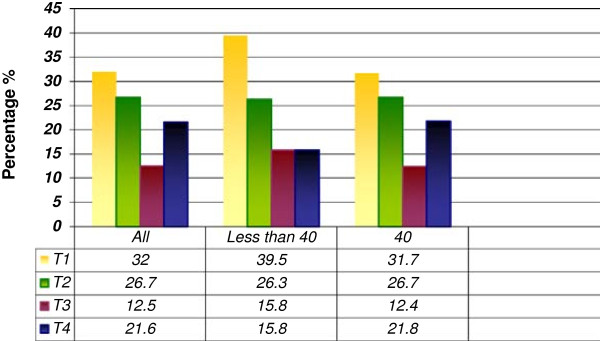
**T-stages (based on T1-T4) by age groups of patients (n = 977).** A comparison of the patients less than 40 years with those older than 40 years shows no significant result using the log-rank test.

As at the time of diagnosis, T_1_ tumours occurred most frequently, accounting for 32.0% of the whole patient population. Males were seen to have larger tumours at presentation than females with a p-value of 0.005 which is highly significant. However, there was no significant difference between the groups of patients less than 40 years and those older than 40 years. (Figures 1 and 2)

### Treatment

Information regarding treatment could not be found for 21 (2.1%) patients and 4 (0.4%) patients received no treatment at all. However, surgery was the only treatment given to 67.5% of the general population, while 14.8% had surgery in combination with chemotherapy or radiotherapy. Only 1.5% had radiotherapy alone, 1.3% had chemotherapy and 0.1% had hormone therapy. In the group of patients less than 40 years, surgery alone was the treatment in 26 (68.4%) patients, surgery in combination with chemotherapy and/or radiotherapy was used for 9 (23.7%) patients and 3 (7.9%) patients had only chemotherapy plus radiotherapy.

### Second primaries, recurrences and metastasis

Secondary tumours developed in 112 patients (11.5%) during the study period, 190 patients (19.4%) had recurrences, 388 patients (39.7%) showed signs of clinical metastasis and in 54 other patients (5.5%), there was distant metastasis.

### Local recurrence-free survival

**Figure 3:**
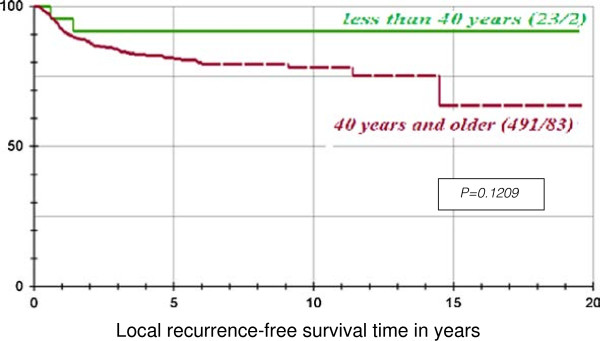
Graphical representation of the local recurrence-free survival time, of the 2 age groups, with the help of the product-limit method of Kaplan-Meier.

Comparison of the local recurrence-free survival for the group less than 40 years and the group above 40 years using the log-rank test yielded no significant result (p = 0.1209). (Figure 3)

### Cause of death and survival analysis

**Figure 4:**
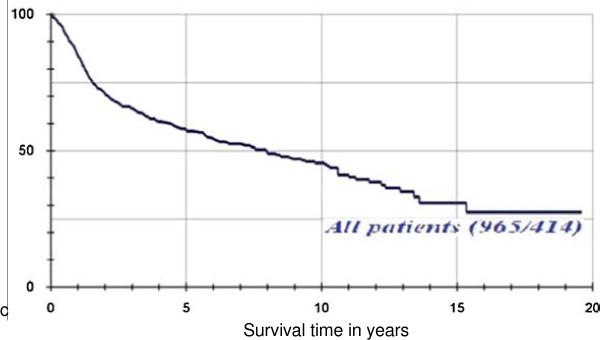
Graphical representation of the survival probability of the entire group, with the help the product-limit method of Kaplan-Meier.

By the end of the study period, 418 patients (42.8%) had died of the tumour, while 559 patients (57.2%) were still alive. A further breakdown revealed that the overall 5-year survival rate was 57.9%, 10-year survival rate was 46.9%, and 15-year survival was 30.9%. (Figure 4)

**Figure 5:**
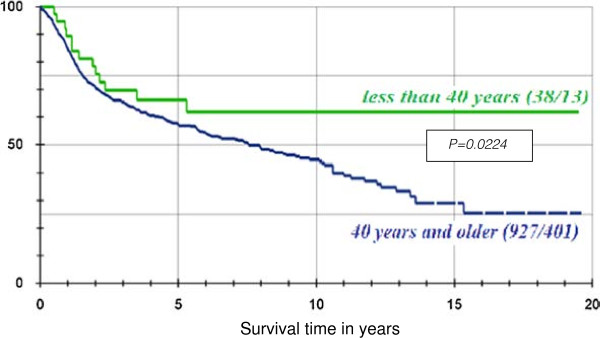
Graphical representation of the survival probability of the 2 age groups, with the help of the product-limit method of Kaplan-Meier.

Among the 38 patients less than 40 years of age, 13 patients (34.2%) had died of the tumour at the end of the study period, while the 25 other patients (65.8%) were still alive or their death was not caused by the tumour. The 5-year survival rate for this group was 66.2%. (Figure 5)

Furthermore, for the group 40 years and older, 401 (42.7%) patients had died of the tumour at the end of the study period, with a 5-year survival rate of 57.6%. (Figure 5)

## Discussion

Many conflicting results have been reported regarding whether age at diagnosis influences prognosis of squamous cell carcinoma of the head and neck region [1,2,4,8-12]. We present in this report our own experience in the management of these young patients over a 20-year period with comparison also to the older patients’ population seen within the same study period. An incidence of 3.9% of SCC of the OC and OP in patients less than 40 years of age found in this study compares favourably with other similar reports, thus confirming the rarity of this tumour in young patients [13,14]. Hart et al. [1] however reported an incidence of 15.75% which is significantly higher than the results found in other literature. The authors noted that their small sample size and the overwhelming young population in attendance to their hospital may have contributed to the high incidence rate.

There is a general agreement in the literature of a higher male preponderance of SCC of the OC and OP in young patients [4,15-18]. A male to female ratio of 3.8:1 observed among the young patient group in our study is a bit higher than a few other reports which gave the gender ratio to be 1.6:1 [15], 1.7:1 [4], 1.9:1 [16], and 2.3:1 [17]. Our result on the other hand is far less than 6.7:1 male to female ratio reported by Cusumano and Persky [18]. Geographic locations and social lifestyles among different world populations might have accounted for these variations.

The tongue is reported as the most common location for head and neck SCC in the young adults [1,2,4,19]. This differs from the findings from the present study in which the tongue is second only to floor of the mouth as the commonest location. However, considering women in this young age group alone, the tongue was by far the preferred site accounting for 50% of the tumour location. This agrees with the findings of Patel et al. [7] who reported an increasing incidence of oral tongue SCC among young white women. Significantly, no tumour was located in the oropharynx of the young patients’ population and this site also accounted only for 1.3% of the tumour location in the general study population.

Oral cavity and oropharyngeal SCC in the young patients has been linked with history of exposure to human papilloma virus (HPV), herpes simplex virus (HSV), human immunodeficiency virus (HIV), and some other viral infective diseases, although their causative role remains to be established [6,19,20]. The fact that even when these young patients indulge in known risk factors of alcohol and smoking, it is for considerably shorter periods compared to the adult counterparts as to induce oral carcinogenesis [3,19] leaves room for further search as to other responsible aetiological factors. We cannot however draw any conclusions from our study as regards risk factor-tumour location association since our retrospective review did not include any of the viral risk factors.

As regards the tumour staging and grading, there was no significant difference between the groups of patients less than 40 years and those above 40 using the log-rank test (Tables 2 and 3, Figure 2). T_1_ and T_2_ tumours were the predominant stages for both groups and majority were moderately differentiated tumours. Mallet et al. [2] in a multi-centre analysis of head and neck SCC in young patients in France also reported that most of their patients presented with T_1_ and T_2_ tumours. But of significance in our report however is the result showing that males presented with larger tumours than females at diagnosis (p = 0.005). This underscores the fact that women generally take better care of their bodies than men and visit the hospitals for check-ups more frequently than men do [21].

There is a general claim by some authors that patients in the younger age group have a more aggressive disease with a higher incidence of local recurrence or regional lymph node involvement after treatment compared with older patients [10,22]. Our comparison of the local recurrence-free survival for the group less than 40 years and the group above 40 years using the log-rank test yielded no significant result (p = 0.1209). The only observed discrepancy in this regard was that the young patients did not show sign of recurrences after 2 years whereas in the older patients, there were recurrences even after 10 years following treatment (Figure 3). This may be expected because drinking and smoking habits are more prevalent in the older age group and hence can result in a significantly increased risk of second and subsequent smoking-related cancers [23].

Our records show that at the end of the study period, 13 (34.2%) out of the 38 patients in the young group had died of the tumour and 401 (42.7%) out of 927 in older patients’ group had also died from the tumour. The overall 5-year survival was found to be 66.2% and 57.2% in patients less than 40 years and those above 40 years respectively. This was found to be statistically significant when compared using the log-rank test (p = 0.0224). Before we jump to the conclusion that younger patients have a better prognosis based on the above findings, we should remember the effect of comorbidities on head and neck cancer patients which affects the older patients more [24]. Patients with higher comorbid burden are likely to die earlier due to concurrent illness compared to those who are otherwise free of diseases apart from head and neck cancer [25]. This will therefore affect the overall survival of the patient [26].

## Conclusions

Our review revealed that patients less than 40 years with SCC of OC and OP have a comparable histopathologic features and clinical course with those older than 40 years of age. Their local recurrence-free survival also shows no statistical significant difference, however, the 5-year overall survival is better in the young adult group than in the older patients’ group. This goes to show that there may not be need for a more aggressive treatment for young patients with SCC of the OC and OP as is being advocated in some literature.

## Competing interests

The authors declare that they have no competing interests.

## Authors’ contributions

SEU, AME made substantial contributions to conception and design of the manuscript as well as data acquisition. MR has been involved in drafting the manuscript. WG was involved in data acquisition and statistical analysis. NCG was involved in revising the manuscript. All authors read and approved the final manuscript.
